# Associations between genetic variants in immunoregulatory genes and risk of non-Hodgkin lymphoma in a Chinese population

**DOI:** 10.18632/oncotarget.14426

**Published:** 2017-01-02

**Authors:** Xibiao Ye, Kaiqiong Zhao, Cuie Wu, Pingzhao Hu, Hua Fu

**Affiliations:** ^1^ Department of Community Health Sciences, College of Medicine, Faculty of Health Sciences, University of Manitoba, Canada; ^2^ Vaccine and Drug Evaluation Centre, University of Manitoba, Canada; ^3^ Centre for Healthcare Innovation, University of Manitoba, Winnipeg, Manitoba, Canada; ^4^ Department of Biochemistry and Medical Genetics, College of Medicine, Faculty of Health Sciences, University of Manitoba, Canada; ^5^ School of Public Health, Fudan University, Shanghai, China

**Keywords:** non-Hodgkin lymphoma, genetic susceptibility, single nucleotide polymorphism

## Abstract

We undertook a hospital-based case-control study to examine the associations between single nucleotide polymorphisms (SNPs) in selected immunoregulatory genes and non-Hodgkin lymphoma (NHL) risk in a Chinese population. One hundred and sixty-nine NHL patients diagnosed according to the World Health Organization (WHO) 2001 standard and 421 controls were recruited. Nine SNPs in three genes (*IL-10, IL-1RN, and TNF-α*) were selected based on predicted functions and previous study findings. Genetic association analysis was performed using the Cochran-Armitage trend test and multiple logistic regression. Four SNPs were associated with an increased risk of overall NHL: odds ratio per minor allele [OR_per-minor-allele_] and 95% confidence interval [CI] were 2.64 (1.75-3.98) for *IL-10* rs1800893, 2.67 (1.72-4.16) for *IL-1RN* rs4251961, 1.80 (1.24-2.63) for *TNF- α* rs1800630, and 1.55 (1.02-2.37) for *TNF- α* rs2229094. These SNPs were also associated with an increased risk of diffuse large B-cell lymphoma (DLBCL). In addition, another SNP (*TNF- α* rs1041981) was associated with an increased risk of DLBCL (OR_per-minor-allele_=1.73, 95% CI 1.14-2.61). The findings provide evidence on the role of these immunoregulatory gene variants in NHL etiology.

## INTRODUCTION

Non-Hodgkin lymphoma (NHL) is one of the most common cancers. Overall NHL incidence has been rising for several decades in industrialized countries and then started to level off in the mid-1990s.[1, 2] During 1973-2010, overall NHL incidence increased by 1.64% (from 3.43 to 5.05 per 100,000 person-years) in males and by 2.50% (from 1.77 to 3.65 per 100,000 person-years) in females in Shanghai, China.[3] However, age-standardized NHL mortality rate in China [4] has not declined over time as observed in other countries since the 1990s.[1, 5]

The causes of most NHL cases are poorly understood. There are significant differences in geographical distributions of NHL incidence,[6] probably due to differences in risk factors, genetic susceptibility, and diagnosis/classification. Immunosuppression plays an important role in lymphomagenesis [7] and certain immunodeficiency disorders including congenital immunodeficiency disorders, acquired immunodeficiency syndrome (AIDS), and autoimmune disorders (e.g., rheumatoid arthritis and Sjögren’s syndrome) increase the risk of developing NHL.[8] Early studies found that variants in genes involved in immunoregulatory pathways were associated with an increased or reduced risk of NHL.[9] A meta-analysis showed that *TNF-α* rs1800629 was associated with an increased risk of overall NHL and diffuse large B-cell lymphoma (DLBCL, a major NHL subtype).[10] After pooling three studies together, Hosgood III and colleagues found an association between *IL-1RN* rs2637988 and increased risk of overall NHL.[11] However, studies on these variants have been mainly conducted among subjects of European ancestry and have rarely been replicated in other populations. In this study, we examined the associations between immunoregulatory gene variants and NHL risk in a Chinese population.

## RESULTS

**Figure 1:**
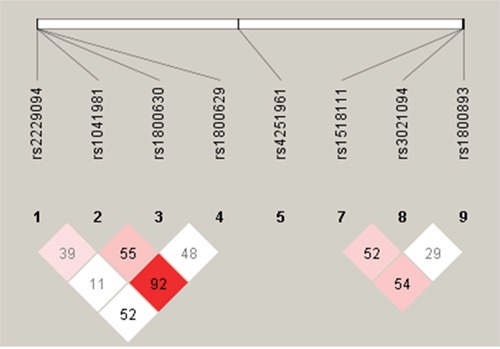
LD plot of the 8 SNPs in genes IL-10 and TNF-α Notes: LD plot showing LD patterns among the 8 SNPs in genes *IL-10* and *TNF-α* genotyped in the 590 samples. The LD between the SNPs is measured as *r*^2^ and shown (×100) in the diamond at the intersection of the diagonals from each SNP. *r*^2^ = 0 is shown as white, 0< *r*^2^<1 is shown in pink and *r*^2^ =1 is shown in red. The analysis track at the top shows the SNPs according to chromosomal location.

Cases and controls were similar regarding the distributions of sex, age, marriage status, and alcohol consumption. But cases tended to be less educated, less likely to be overweight, and more likely to be a smoker (past or current) or have a family history of cancer (Table 1). All nine SNPs had a minor allele frequency larger than 5%. Eight of the nine SNPs had a Hardy-Weinberg Equilibrium (HWE) test *P* value in controls larger than 0.01. *IL-4* rs2243267 had a HWE test *P* value in controls smaller than 1 × 10^-4^, which was removed from the association analysis. Table 2 shows that five SNPs were associated with an increased risk of overall NHL or B-cell lymphomas (particularly DLBCL). *IL-10* rs1800893 was associated with an increased risk of overall NHL (per-minor-allele odds ratio [OR_per-minor-allele_]=2.64, 95% confidence interval [CI] 1.75-3.98), B-cell lymphomas (OR_per-minor-allele_=2.75, 95% CI 1.79-4.23), and DLBCL (OR_per-minor-allele_=3.03, 95% 1.85-5.01). Similar associations were found between *IL-1RN* rs4251961 and an increased risk of overall NHL (OR_per-minor-allele_ =2.67, 95% CI 1.72-4.16), B-cell lymphomas (OR_per-minor-allele_=2.75, 95% CI 1.73-4.36), and DLBCL (OR_per-minor-allele_=3.39, 95% CI 1.99-5.97). Three *TNF-α* SNPs were associated with an increased NHL risk: rs1041981 was associated with an increased risk of B-cell lymphoma (OR_per-minor-allele_=1.45, 95% CI 1.02-2.05), particularly DLBCL (OR_per-minor-allele_=1.73, 95% CI 1.14-2.61); rs1800630 was associated with a higher risk of overall NHL (OR_per-minor-allele_ for overall NHL=1.80, 95% CI 1.24-2.63), B-cell lymphomas (OR_per-minor-allele_ =1.76, 95% CI 1.19-2.61), and DLBCL (OR_per-minor-allele_=1.88, 95% CI 1.17-3.03); rs2229094 was associated with an increased risk of overall NHL (OR_per-minor-allele_ =1.55, 95% CI 1.02-2.37) and DLBCL (OR_per-minor-allele_=1.83, 95% CI 1.09-3.08). No statistically significant associations were found for the other three SNPs (*IL-10* rs1518111, *IL-10* rs3021094, and *TNF-α* rs1800629). We obtained linkage disequilibrium (LD) patterns of all 8 SNPs in the genes *IL-10* and *TNF-α* using Haploview (Figure 1). As shown in Figure 1, the significant SNPs (*TNF-α* rs1800630 and *TNF-α* rs1041981) had a relatively high correlation (0.55) and were in fairly high LD with each other. The same trend was observed for the significant SNPs *TNF-α* rs1041981 and *TNF-α* rs2229094. We also found that *IL-10* rs1518111, *IL-10* rs3021094, and *IL-10* rs1800893 had relatively high LD with each other.

**Table 1:** Characteristics of NHL Cases and Controls in Shanghai, China

Characteristics	Controls (n=421)	Cases (n=169)	Total (n=590)
Gender			
Female	258 (61.3)	103 (60.9)	361 (61.2)
Male	163 (38.7)	66 (39.1)	229 (38.8)
Age (year)			
<50	106 (25.2)	50 (29.6)	156 (26.4)
50-65	186 (44.2)	77 (45.6)	263 (44.6)
65+	129 (30.6)	42 (24.9)	171 (29.0)
Mean (SD)	57.9 (13.7)	55.1 (12.9)	57.1 (13.5)
Education*			
Less than secondary school	73 (17.3)	144 (85.2)	217 (36.8)
Secondary school	268 (63.7)	25 (14.8)	293 (49.7)
College or university	80 (19.0)		80 (14.5)
Marriage			
Never	10 (2.4)	7 (4.1)	17 (2.9)
Current	361 (85.8)	154 (91.1)	515 (87.3)
Divorced/Widowed	33 (7.8)	8 (4.7)	18 (3.1)
Missing	17 (4.0)		17 (2.9)
BMI* (kg/m^2^)			
<18.5	201 (47.7)	104 (61.5)	305 (51.7)
18.5-24	11 (2.6)	10 (5.9)	21 (3.6)
24-28	165 (39.2)	46 (27.2)	211 (35.7)
28+	44 (10.5)	9 (5.3)	53 (8.9)
Mean (SD)	24.3 (6.7)	22.9 (3.1)	23.9 (5.9)
Smoking*			
Never	267 (63.4)	94 (55.6)	361 (61.2)
Past	37 (8.8)	35 (20.7)	72 (12.2)
Current	116 (27.6)	40 (23.7)	156 (26.4)
Missing	1 (0.2)		1 (0.2)
Alcohol			
No	328 (77.9)	128 (75.7)	456 (77.3)
Yes	91 (21.6)	41 (24.3)	132 (22.4)
Missing	2 (0.5)		2 (0.3)
Family history of cancer*			
No	322 (76.5)	51 (30.2)	373 (63.2)
Yes	98 (23.3)	118 (69.8)	216 (36.6)
Missing	1 (0.2)		1 (0.2)

Note: The number in bracket is proportion (%) of the total number in controls; *, P<0.001 for the comparisons between cases and controls.

**Table 2:** Associations between immunity gene SNPs and NHL risk

Gene	SNP	Minor allele	Alternative allele	Control (%)	Overall NHL				B-Cell Lymphomas				DLBCL			
					AFF (%)	OR_per-minor-allele_ (95% CI)≠	P_CA_BH_	P_logistic_BH_	AFF (%)*	OR_per-minor-allele_ (95% CI)≠	P_CA_BH_	P_logistic_BH_	AFF (%)*	OR_per-minor-allele_ (95% CI)≠	P_CA_BH_	P_logistic_BH_
*IL-10*	rs1518111	G	A	12/46/36	9/53/34	1.16 (0.83-1.60)	0.776	0.492	8/51/37	1.08 (0.77-1.53)	0.710	0.677	10/48/37	1.11 (0.75-1.67)	0.667	0.598
*IL-10*	rs1800893	T	C	1/30/62	2/53/40	2.64 (1.75-3.98)	<0.0001	<0.0001	1/55/40	2.75 (1.79-4.23)	<0.0001	<0.0001	2/55/37	3.03 (1.83-5.01)	<0.0001	<0.0001
*IL-10*	rs3021094	G	T	17/49/24	14/53/24	0.86 (0.62-1.20)	0.648	0.492	15/55/20	0.96 (0.68-1.36)	0.860	0.861	17/60/18	1.12 (0.74-1.68)	0.449	0.598
*IL-1RN*	rs4251961	C	T	1/17/74	4/27/64	2.67 (1.72-4.16)	0.001	<0.0001	4/27/65	2.75 (1.73-4.36)	0.003	0.003	4/31/60	3.39 (1.99-5.79)	<0.0001	<0.0001
*TNF-α*	rs1041981	T	G	17/49/25	19/55/18	1.34 (0.96-1.87)	0.211	0.146	21/55/16	1.45 (1.02-2.05)	0.087	0.087	25/57/12	1.73 (1.14-2.61)	0.021	0.022
	rs1800629	A	G	0/14/76	1/12/79	0.89 (0.49-1.59)	0.659	0.785	1/13/78	1.02 (0.56-1.85)	0.861	0.957	1/10/83	0.79 (0.38-1.67)	0.449	0.598
	rs1800630	A	C	4/24/64	1/46/49	1.80 (1.24-2.63)	0.007	0.006	1/44/51	1.76 (1.19-2.61)	0.028	0.015	0/45/51	1.88 (1.17-3.03)	0.119	0.022
	rs2229094	G	A	2/25/51	2/37/48	1.55 (1.02-2.37)	0.132	0.089	2/37/50	1.52 (0.98-2.36)	0.198	0.113	1/43/47	1.83 (1.09-3.08)	0.129	0.042

* Proportion of samples with the genotypes (rare homozygosity/ heterozygosity /common homozygosity) in the given group;

≠ Calculated from multiple logistic regression model adjusted for sex, age, education, family history of NHL, smoking, environmental exposures, and body mass index (BMI).

SNP, single nucleotide polymorphism; AFF, number of cases with rare homozygous, heterozygous and common homozygous genotypes; OR_per-minor-allele_, per-minor-allele odds ratio; 95% CI, 95% confidence interval; PCA_BH, p value of Cochran-Armitage trend test (univariate analysis) corrected for multiple testing using Benjamini and Hochberg (BH) approach; Plogistic_logistic_BH_, p value of multiple logistic regression model corrected for multiple testing using BH approach; DLBCL, diffuse large B-cell lymphomas; NHL, non-Hodgkin lymphoma.

## DISCUSSION

In this study, we found associations between five immunoregulatory gene SNPs and a higher NHL risk: four SNPs (*IL-10* rs1800893, *IL-1RN* rs4251961, *TNF-α* rs1800630, and *TNF-α* rs2229094) were associated with an increased risk of overall NHL and DLBCL, while *TNF- α* rs1041981was associated with an increased risk of DLBCL only.

Cytokines including tumor necrosis factor (*TNF*) play a role in lymphomagenesis and genetic variants that upregulate or downregulate the production of cytokines may relate to NHL risk.[12] Previous studies have shown an association between *TNF-α* rs1800629 and an increased risk of NHL (particularly subtypes DLBCL, marginal zone lymphoma, and T-cell lymphoma) when ethnicity was not considered.[9, 13] However, a pooled analysis showed that the association between the SNP and NHL risk varied by ethnicity: *TNF-α* rs1800629 minor allele A (genotypes GA/AA) was associated with a reduced risk of NHL in Asian participants living in Australia (OR=0.52, 95% CI 0.32-0.85, compared to genotype GG), but was associated with an increased NHL risk in African Americans and Hispanic Whites.[10] The ethnic difference was further verified by a recent meta-analysis.[14] The inverse association was confirmed in a recent study in Chinese and Korean populations,[15] where rs1800629 minor allele A was associated with 20% lower risk for NHL. The inverse association was not observed in the present study and this might relate to the small sample size and the difference in study methods (e.g., only Han Chinese living in China were included in the present study).

Another SNP (*TNF- α* rs1800630 minor allele A) was associated with an increased risk of NHL in the present study and this is consistent with the findings of the other two studies in Asia.[15, 16] The present study and the study by Gu [16] included Han Chinese only and found a 80% higher risk for NHL among *TNF-α* rs1800630 minor allele A carriers. Another study involving a mixed population of Han Chinese and Koreans showed a 19% increased NHL risk among minor allele A carriers.[15] In an earlier study in Australia, this SNP was also associated with a higher risk for NHL particularly for DLBCL.[17] Two *TNF- α* SNPs (rs1041981 and rs2229094) examined in the present study have been rarely investigated previously. *TNF-α* rs1041981 was associated with a higher risk of AIDS-related NHL without considering a CD4 count change, but the association did not hold after adjusting for the rate of the change in CD4 count.[18] In the present study, both *TNF-α* rs1041981 and *TNF-α* rs2229094 minor alleles are associated with a higher risk of NHL, particularly DLBCL, and the findings need to be tested in a larger study.

*IL-10*, also known as human cytokine synthesis inhibitory factor, is a cytokine with multiple effects in immunoregulation and anti-inflammation. We found that *IL-10* rs1800893 was associated with an increased risk of NHL, but the association was not observed in a previous study in Germany.[19] Two other common *IL-10* SNPs (rs1800890 and rs1800896) were associated with an increased risk of NHL and its major subtype DLBCL in Hispanic Whites, but not in Asians or African Americans in previous studies [10] (therefore they were not examined in the present study). A more recent study confirmed that there was no a significant association between *IL-10* rs1800896 and NHL risk in Chinese.[20]

The protein encoded by *IL-1RN* gene inhibits the activities of interleukin 1 α and β and modulates a variety of interleukin 1 related immune and inflammatory responses. *IL-1RN* rs4251961 was found to be associated with an increased risk of cancers e.g. colorectal cancer,[21] but our study for the first time reports its association with an increased risk of NHL. Other *IL-1RN* SNPs (rs2637988 and rs454078) were also associated with an increased NHL risk in previous studies,[13, 15] but were not included in the present analysis.

Previous genome-wide association studies (GWAS) have identified a few SNPs that were associated with an increased risk of overall NHL,[22] chronic lymphocytic leukemia,[23, 24] and follicular lymphoma,[22, 23, 25, 26] in populations of European ancestry. Fewer studies have been conducted in Asian populations. In Japan, rs751837 was associated with a higher risk of DLBCL.[27] However, none of these GWAS associations have been replicated in other populations. Two recent Chinese studies have identified SNPs associated with an increased risk of B-cell NHL (rs6773854 located between *BCL6* and *LPP* genes on chromosome 3q27, *IRF4* rs872071, and rs2647012 in HLA-region)[28, 29] and T-cell NHL (*ACOXL* rs17483466 and *IRF4* rs872071),[28] but none of these SNPs are in immunoregulatory genes.

NHL is a cancer of immune system and immune deficiency is one of a few well-established risk factors. There is also evidence on the role of other immune diseases including atopy and autoimmune diseases in NHL risk.[7] Single nucleotide variants may change immunoregulatory gene functions and therefore influence NHL risk through the immunoregulation pathways. This hypothesis is supported by the findings from the present study and previous studies despite the heterogeneity in findings.[10, 11, 30] GWAS studies have also identified a few immune gene SNPs associated with an increased risk of NHL or specific NHL subtypes, but overall the findings were inconsistent and suggestive.

There are several limitations in the present study. First, the observed associations may not be interpreted as causal relationships due to the nature of case-control design. It is likely that the findings have been biased as controls were more likely residents from other provinces. Information on covariates was collected retrospectively via an interview, so recall bias and misclassification could not be ruled out. The small sample size also limited the ability to analyze other NHL subtypes (e.g., follicular lymphoma, marginal zone lymphoma, T-cell lymphoma). The analysis was limited to a small number of candidate SNPs selected based on previous epidemiologic study findings and has not tested SNPs associated with NHL risk in more recent studies. Despite that, the present study supports that immunoregulatory gene variants play a role in NHL risk. Further research on the racial/ethnic difference in associations between genetic variants and NHL risk as well as the functional experiment analysis of the associations may advance the understanding of the etiology of NHL.

## MATERIALS AND METHODS

Eligible cases were NHL patients diagnosed between 2003 and 2008 according to the WHO 2001 classification standard and aged 18 or older, as described in detail previously.[31] Patients with a provisional diagnosis of NHL from the participating hospitals were referred to a leukemia and lymphoma diagnosis and research laboratory located at Fudan University, China. Peripheral blood, bone marrow aspirates, tissue and core biopsies of patients were collected in conjunction with diagnostic procedures and were sent to the laboratory for morphological, immunological, cytogenetic, and molecular genetic analyses.[32, 33] Controls were randomly selected from patients in any other wards from the same hospitals matched on sex and age group frequency. Those with cancers or non-malignant lymphatic or hematopoietic diseases, or having family connections to the cases were excluded. One hundred and sixty-nine cases and 421 controls were included into the study. Each patient completed a questionnaire containing questions regarding demographics, family history, lifestyles, occupational and environmental exposures. This project has been approved by the Ethical Review Committee of Fudan University in accordance with the International Ethical Guidelines for Biomedical Research Involving Human Subjects (2002).[34] Informed consent was obtained from all individual participants in the study. Genes were selected based on two criteria: they are involved in immunoregulation and were associated with NHL risk in previous studies, using the method by Xu.[35] A total of nine SNPs in four genes were selected, including *IL-10* (rs1518111, rs1800893, and rs3021094), *IL-1RN* (rs4251961), *IL-4* (rs2243267), and *TNF-α* (rs1041981, rs2229094, rs1800629, and rs1800630). Genotyping was conducted using the real-time polymerase chain reaction method on an ABI 7900HT sequence detection system at the State Key Laboratory of Genetics at Fudan University.[36] For quality control, 10% of samples were randomly selected and re-genotyped. The concordance rates for replicated samples were 100%.

Chi-square test and *t*-test were used for comparisons of categorical and continuous variables between cases and controls. To test the associations between SNPs and NHL risk, we first performed the Cochran-Armitage trend test using only genotype data.[37] Multiple logistic regression models were then applied based on a genetic additive model. OR_per-minor-allele_ and 95% CI were estimated using major allele as a reference. The models were fitted for overall NHL, B-cell lymphomas, and diffuse large B-cell lymphoma (DLBCL) separately, but not for T-cell lymphoma patients due to the small sample size. As *a prior*,[8] we included covariates sex, age, education, family history of NHL, smoking status, environmental exposures (i.e., exposed to one or more of substances including benzene, solvents, metals, agrichemicals, or pesticides), body mass index (BMI) and hair dye use in the initial model. The inclusion of a variable into the final model was determined using the stepwise approach (“stepAIC” function in MASS R package).[38] Covariates sex, age, education, family history of NHL, smoking, environmental exposures, and BMI were controlled in the final logistic regression model.[37] If a sample with missing data on any of the variables in the regression model, the sample was excluded from the model analysis. P values were adjusted using the Benjamini and Hochberg multiple testing procedure.[39] All statistical analyses were undertaken in statistical software R and PLINK. [37] Haplotype analysis was performed using Haploview.[40]
